# MP-12 virus containing the clone 13 deletion in the NSs gene prevents lethal disease when administered after Rift Valley fever virus infection in hamsters

**DOI:** 10.3389/fmicb.2015.00651

**Published:** 2015-06-29

**Authors:** Brian B. Gowen, Jonna B. Westover, Eric J. Sefing, Kevin W. Bailey, Shoko Nishiyama, Luci Wandersee, Dionna Scharton, Kie-Hoon Jung, Tetsuro Ikegami

**Affiliations:** ^1^Department of Animal, Dairy, and Veterinary Sciences, Utah State University, Logan, UT, USA; ^2^Institute for Antiviral Research, Utah State University, Logan, UT, USA; ^3^School of Veterinary Medicine, Utah State University, Logan, UT, USA; ^4^Department of Pathology, The University of Texas Medical Branch, Galveston, TX, USA; ^5^Sealy Center for Vaccine Development, The University of Texas Medical Branch, Galveston, TX, USA; ^6^Center for Biodefense and Emerging Infectious Diseases, The University of Texas Medical Branch, Galveston, TX, USA

**Keywords:** Rift Valley fever virus, phlebovirus, viral hemorrhagic fever, vaccine, post-exposure

## Abstract

Rift Valley fever virus (RVFV; *Bunyaviridae, Phlebovirus*) causes a range of illnesses that include retinitis, fulminant hepatitis, neurologic disease, and hemorrhagic fever. In hospitalized individuals, case fatality rates can be as high as 10–20%. There are no vaccines or antivirals approved for human use to prevent or treat severe RVFV infections. We previously tested the efficacy of the MP-12 vaccine strain and related variants with NSs truncations as a post-exposure prophylaxis in mice infected with wild-type pathogenic RVFV strain ZH501. Post-exposure efficacy of the rMP12-C13type, a recombinant MP-12 vaccine virus which encodes an in-frame truncation removing 69% of the NSs protein, resulted in 30% survival when administering the virus within 30 min of subcutaneous ZH501 challenge in mice, while the parental MP-12 virus conferred no protection by post-exposure vaccination. Here, we demonstrate uniform protection of hamsters by post-exposure vaccination with rMP12-C13type administered 6 h post-ZH501 infection while no efficacy was observed with the parental MP-12 virus. Notably, both the MP-12 and rMP12-C13type viruses were highly effective (100% protection) when administered 21 days prior to challenge. In a subsequent study delaying vaccination until 8, 12, and 24 h post-RVFV exposure, we observed 80, 70, and 30% survival, respectively. Our findings indicate that the rapid protective innate immune response elicited by rMP12-C13type may be due to the truncated NSs protein, suggesting that the resulting functional inactivation of NSs plays an important role in the observed post-exposure efficacy. Taken together, the data demonstrate that post-exposure vaccination with rMP12-C13type is effective in limiting ZH501 replication and associated disease in standard pre-exposure vaccination and post-challenge treatment models of RVFV infection, and suggest an extended post-exposure prophylaxis window beyond that initially observed in mice.

## Introduction

Rift Valley fever virus (RVFV) causes a devastating mosquito-borne disease in ruminants throughout Africa and the Arabian Peninsula, which results in abortion storms and high mortality among young animals (Bird et al., 2009). RVFV also infects humans via mosquito bite or exposure to animal tissues during the processing or handling of infected animals. In humans, the majority of individuals infected with RVFV will develop clinical signs of an acute febrile illness characterized by fever, malaise, and headaches with most patients recovering without serious complications (Laughlin et al., 1979; McIntosh et al., 1980; Madani et al., 2003). However, some cases can progress to a fatal hemorrhagic fever syndrome, encephalitis, hepatitis, renal failure, or retinitis that can lead to blindness.

A member of the *Bunyaviridae* family (genus *Phlebovirus)*, the enveloped RVFV possesses a tri-partite negative-sense RNA genome that encodes seven proteins (Ikegami, 2012). The large (L) segment encodes the RNA-dependent RNA-polymerase. The medium (M) segment encodes the surface glycoproteins Gn and Gc, the non-structural protein NSm, and the 78-kD protein. The small (S) segment has an ambisense configuration and encodes the nucleocapsid protein, N, and an additional non-structural protein, NSs, which is transcribed from antigenomic-sense viral RNA. NSs is considered to be a major virulence factor in the evasion of the host cell antiviral defenses (Ikegami and Makino, 2011). NSs inhibits host transcription and the induction of interferon (IFN)-β through specific interactions with host cell transcription factors (Le May et al., 2004, 2008; Kalveram et al., 2011; Kainulainen et al., 2014). The NSs protein also promotes the degradation of double-stranded RNA-dependent protein kinase, PKR (Habjan et al., 2009; Ikegami et al., 2009), further interfering with host antiviral defenses by inactivating an important cytosolic sensor of viral replication intermediates (Dauber and Wolff, 2009).

The antiviral drug, ribavirin, may be considered for treatment of RVF, but its use is associated with toxicity and has not been evaluated as a post-exposure prophylaxis (Borio et al., 2002). Although many vaccine strategies have been developed to combat RVF, none are approved for human use to prevent or treat infections (Kortekaas, 2014). The modified live MP-12 RVFV vaccine strain was developed by serial mutagenesis of the ZH548 strain (Caplen et al., 1985), with the partial attenuations mapped to the S-, M-, and L-segments (Ikegami et al., 2015). It has been extensively studied to assess safety and immunogenicity (Morrill et al., 1987, 1991, 1997a,b; Hunter et al., 2002; Morrill and Peters, 2003; Wilson et al., 2014). We have previously described the use of a recombinant MP-12 modified vaccine virus, rMP12-C13type, which contains an inactivating deletion in the NSs gene based on the naturally attenuated RVFV clone 13 vaccine candidate, (Muller et al., 1995; Dungu et al., 2010; von Teichman et al., 2011), as a potential vaccine and post-exposure intervention in mouse models of RVFV infection (Lihoradova et al., 2012; Gowen et al., 2013). In the present study, we demonstrate significantly enhanced prophylactic capacity of the rMP12-C13type vaccine lacking functional NSs in a more rapidly progressing hamster model of RVF disease and shed light on species variations in the context of post-exposure vaccine efficacy and therapeutic window in rodent infection models.

## Materials and Methods

### Ethics Statement

All animal procedures complied with USDA guidelines and were conducted at the AAALAC-accredited vivarium in the BioInnovations Building at the Utah State University Innovation Campus under protocol 2011, approved by the Utah State University Institutional Animal Care and Use Committee.

### Animals

Female Syrian golden hamsters were obtained from Charles River Laboratories (Wilmington, MA, USA) and quarantined for 72 h prior to challenge or vaccination and fed Harlan Lab Block and tap water *ad libitum*.

### Attenuated Vaccine Viruses

The infectious clone of RVFV, strain ZH501, was obtained from Dr. Stuart Nichol (CDC, Atlanta, GA, USA). The virus stock [1.1 × 10^8^ plaque-forming units (PFU)/ml]; BSR-T7/5, VeroE6-1, Vero-1) used was from a clarified cell culture lysate preparation. The virus stock was diluted in sterile minimum essential media (MEM) and inoculated by either subcutaneous (s.c.) injection (ventral, right side of abdomen) or intranasal (i.n.) instillation (under isoflurane anesthesia). The MP-12 vaccine strain of RVFV was obtained from Dr. Robert Tesh (World Reference Center for Emerging Viruses and Arboviruses, University of Texas Medical Branch, Galveston, TX, USA). The reverse genetics system and rescue of the rMP-12 and rMP12-C13type recombinant vaccine virus strains have been previously described in detail (Ikegami et al., 2006; Kalveram et al., 2011). The recombinant vaccine virus stocks did not contain defective interfering viral RNAs by Northern blot analysis. All vaccine strains were diluted in sterile phosphate buffered saline (PBS) and 0.1 ml was administered s.c. (ventral, left side of abdomen).

### Efficacy of Pre- and Post-exposure Vaccination with rMP12-C13type and MP-12 in Hamsters Challenged s.c. with RVFV ZH501

Five-week-old hamsters were weighed 21 days prior to infection and grouped so that the average weight per group (*n* = 10 for treatment and placebo groups, *n* = 3 the sham-infected group) across the entire experiment varied by less than 5 g. Animals in each group were treated once with PBS placebo or 1 × 10^5^ PFU of MP-12 or rMP12-C13type by s.c. injection 21 days prior or 30 min, 2 or 6 h post-challenge with 30 PFU of RVFV. The hamsters were observed for 21 days for morbidity and mortality following RVFV ZH501 infection.

A second efficacy study was conducted in which the vaccination was administered 8, 12, and 24 h after RVFV ZH501 infection. For this study, 7-week-old hamsters were weighed on the day of infection and grouped so that the average weight per group (*n* = 14 for treatment and placebo groups, *n* = 6 the sham-infected group) across the entire experiment varied by less than 2 g. Animals were challenged s.c. with 30 PFU of RVFV ZH501 followed by a single treatment of 1 × 10^5^ PFU of rMP12-C13type by s.c. injection at either 8, 12, or 24 h post-virus challenge. For comparison, groups of infected animals received the MP-12 virus or the PBS placebo by s.c. treatment at the 8 h post-challenge time. Four animals from each treatment group were sacrificed on day 2 of infection for analysis of viral titers. The remaining animals were observed 28 days for morbidity and mortality.

### Efficacy of Vaccination with rMP12-C13type and MP-12 Post-RVFV i.n. Infection

The post-exposure efficacy of rMP12-C13type vaccine was also evaluated in the i.n. challenge hamster model. Seven-week-old hamsters were weighed on the day of infection and grouped so that the average weight per group (*n* = 10) across the entire experiment varied by less than 6 g. Following i.n. instillation of 1 × 10^5^ PFU of RVFV ZH501, animals in each group were treated once with PBS placebo or 1 × 10^5^ PFU of either rMP12-C13type or rMP-12 by s.c. injection at either 6 or 24 h post-challenge. The animals were observed 21 days for morbidity and mortality.

### Tissue and Serum Virus Titers

Virus titers were assayed using an infectious cell culture assay as previously described (Gowen et al., 2007). Briefly, tissue samples were homogenized in a fixed volume of MEM and the clarified homogenates and serum were serially diluted and added to quadruplicate wells of Vero 76 cell monolayers in 96-well microplates. The viral cytopathic effect (CPE) was determined 7 days post-infection and the 50% endpoints were calculated as described (Reed and Muench, 1938). The detection limit for virus in serum was 1.49 log_10_ 50% cell culture infectious dose (CCID_50_)/ml and in tissues the lower limit of virus detection was 1.97 log_10_ CCID_50_/g of tissue.

### Measurement of RNA Copy Numbers of RVFV RNA and IFN-β mRNA

To evaluate the induction of type I IFN in hamsters by the recombinant vaccine viruses, animals were inoculated in the left hind footpad by s.c. injection of 0.1 ml PBS solution containing 1 × 10^5^ PFU of rMP-12, rMP12-C13type or PBS (mock-inoculated controls). Popliteal lymph nodes (LNs) were harvested at 24 h post-inoculation and preserved in TRI Reagent (Sigma-Aldrich, St. Louis, MO, USA). Total RNA was extracted from popliteal LNs and RNA concentrations were measured using the Qubit 2.0 Fluorometer (Life Technologies, Grand Island, NY, USA). First strand cDNA was synthesized from 100 ng total RNA using iScript reverse transcriptase (Bio-Rad, Hercules, CA, USA) and a QX100 Droplet Digital PCR System (Bio-Rad) was used to measure RVFV S RNA and hamster IFN-β mRNA expression according to the manufacturer’s instructions. The Taqman PCR reaction was performed as follows: initial heating at 95°C for 10 min, 40 cycles of 30 s at 94°C and 1 min at 60°C, and the final step at 98°C for 10 min.

RVFV S RNA was detected by amplification of the N gene sequence with forward primer (5′-GGC TGG CTG GAC ATG C-3′), reverse primer (5′-AGT GAC AGG AAG CCA CTC A-3′), and probe (5′-HEX-CAG GCT TTG GTC GTC TTG AG-BHQ1-3′) designed for the parental MP-12 virus. Because the sequence of IFN-β mRNA for Syrian golden hamsters (*Mesocricetus auratus*) was not available, we cloned a partial IFN-β mRNA sequence for the Taqman probe design. For the detection of hamster IFN-β, forward primer (5′-ACC CTA AAG GAA GTG CCA GA-3′), reverse primer (5′-CCA GCT GCC AGT AAT AGC TC-3′), and probe (5′-HEX-AGT TTG ACT ACA AGG ATT AGC TTG AA-BHQ1-3′) were designed. Because the assay also detected the IFN-β gene in genomic DNA as indicated by a positive signal in the minus-reverse transcriptase (-RT) control reactions, the IFN-β mRNA copy number was calculated by subtraction of the genomic IFN-β signal from the total signal in RT-containing reactions.

### Statistical Analysis

The Mantel-Cox log-rank test was used for analysis of Kaplan-Meier survival curves. A one-way analysis of variance (ANOVA) with a Tukey *post hoc* test was performed to compare differences in viral titers and RNA copy numbers. All statistical evaluations were done using Prism (GraphPad Software, La Jolla, CA, USA).

## Results

### Post-exposure Vaccination with rMP12-C13type Protects Hamsters from Lethal s.c. RVFV Challenge

**FIGURE 1:**
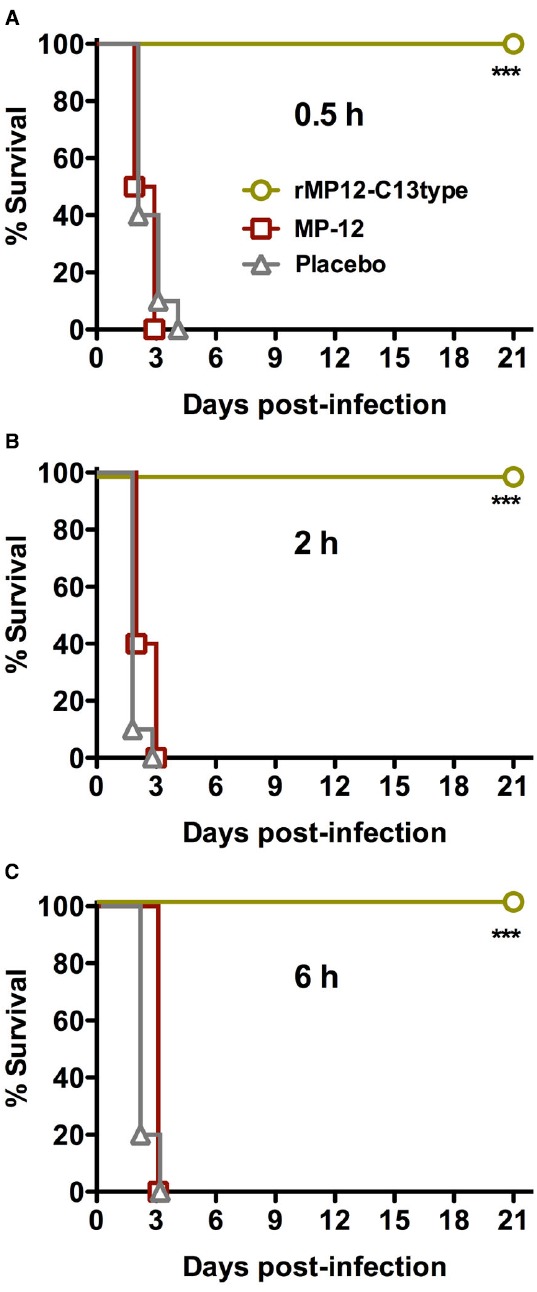
**Survival of Syrian golden hamsters challenged s.c. with RVFV then treated with rMP12-C13type or MP-12 vaccine strains.** Animals in each group (*n* = 10) were treated s.c. with 1 × 10^5^ PFU of rMP12-C13type or MP-12 at (A) 0.5 h, (B) 2 h, or (C) 6 h post-infection. ***P < 0.001 compared to animals that received the placebo.

We first evaluated the efficacy of rMP12-C13type and MP-12 when administered s.c. at 30 min, 2 or 6 h after s.c. challenge with RVFV ZH501. The rMP12-C13type vaccine conferred complete protection against a uniformly lethal RVFV infection at all three post-challenge treatment times (Figure 1). In contrast, the animals that received MP-12 all succumbed to acute infection within 3 days of challenge. Both rMP12-C13type and MP-12 provided full protection when hamsters were vaccinated 21 days prior to challenge (data not shown). The 100% survival rate observed with the post-exposure rMP12-C13type treatment was unexpected based on our previous results in mice demonstrating only partial protection when administered within 30 min of RVFV ZH501 challenge (Gowen et al., 2013).

**FIGURE 2:**
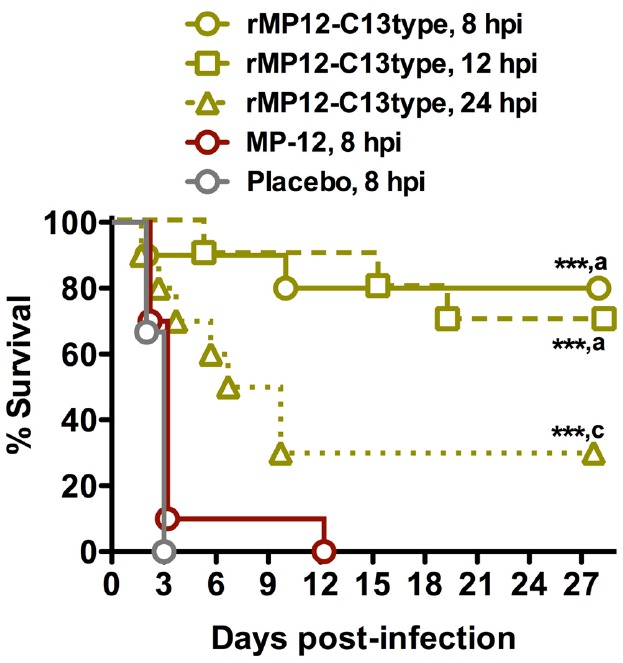
**Survival of hamsters challenged s.c. with RVFV that received post-exposure treatment with rMP12-C13type or MP-12 vaccine strains.** Animals in each group (*n* = 10) were treated s.c. with 1 × 10^5^ PFU of rMP12-C13type, MP-12 or placebo at 8, 12, or 24 h post-infection. ***P < 0.001 compared to animals receiving the placebo. ^a^*P* < 0.001, ^c^*P* < 0.05 compared to animals treated with MP-12.

Because of the dramatic efficacy observed when administered 6 h post-exposure, we next evaluated the dosing of the rMP12-C13type vaccine at 8, 12, or 24 h post-infection (hpi) with s.c. RVFV ZH501. As shown in Figure 2, treatment with the rMP12-C13type virus strain significantly improved survival outcome when compared to the placebo and MP-12-treated animals at all three post-infection timepoints. All of the animals treated at 8 hpi with placebo and all but one of the MP-12-treated animals succumbed to the infection by day 3 with the last animal expiring on day 12. Comparatively, 80% of the 8 hpi rMP12-C13type treatment group, 70% of the 12 hpi group, and 30% of the 24 hpi group survived the lethal challenge, suggesting that the window for post-exposure effectiveness is not far beyond the 24 hpi treatment time. Many of the rMP12-C13type-treated hamsters survived the acute infection, with some animals surviving well into the 3rd week post-infection and most likely succumbing to late-onset neurologic disease previously described in mice treated with rMP12-C13type virus (Gowen et al., 2013) and hamsters protected from acute disease by antivirals (Scharton et al., 2014). Because a detailed analysis of serum, brain, and other tissue virus titers were not performed in the deceased or moribund animals, we were not able to determine if hamsters succumbing beyond day 4 post-infection (the time by which all placebo-treated animals expire) had cleared the systemic infection with virus present only in the brain. However, based on previous work with antiviral treatment of RVFV infection in hamsters (Scharton et al., 2014), we suspect that animals succumbing beyond 7 days post-RVFV challenge had very high viral loads in the brain and associated neurologic disease.

**FIGURE 3:**
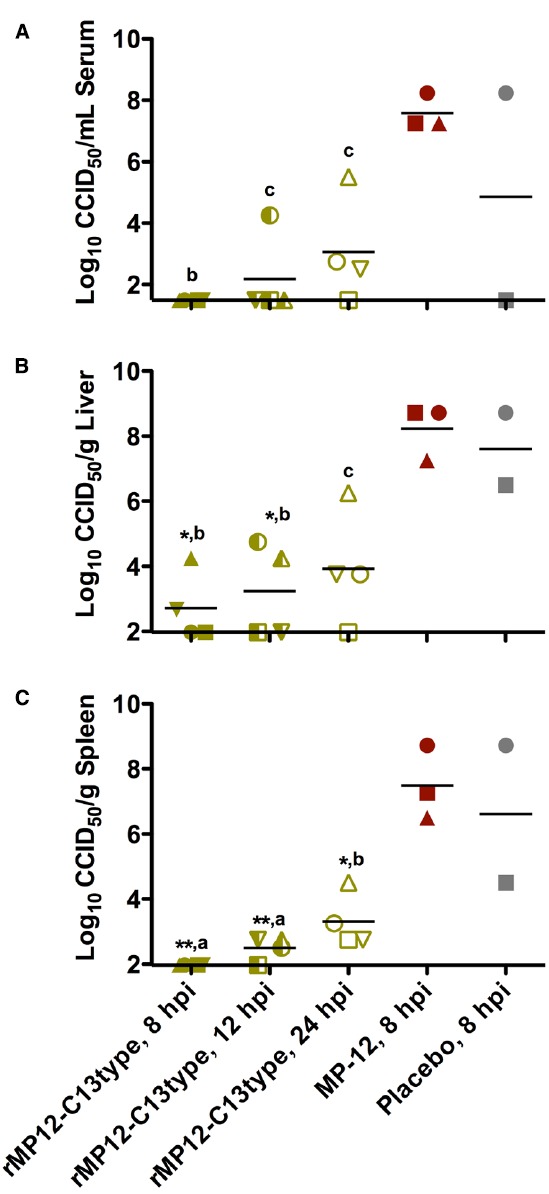
**Analysis of serum and tissue viral titers in RVFV-infected hamsters treated with rMP12-C13type or MP-12 vaccine strains.** Hamsters were treated as described in Figure 2 and up to four animals in each group were sacrificed on day 2 post-infection for analysis of (A) serum, (B) liver, and (C) spleen virus titers. One animal from the MP-12 treatment group and two from the placebo group expired prior to sacrifice on day 2. Unique symbols in each treatment group represent values for the same animal across all parameters. **P < 0.01, *P < 0.05, compared to animals receiving the placebo. ^a^*P* < 0.001, ^b^*P* < 0.01 ^c^*P* < 0.05 compared to animals treated with MP-12.

The effect of the post-exposure treatments on reducing viral titers in subsets of animals sacrificed on day 2 post-infection is shown in Figure 3. Treatment with rMP12-C13type significantly reduced day 2 viral titers at all three timepoints in serum and tissues compared to the MP-12 treatment at 8 hpi. The decrease in viral loads correlated with the time of rMP12-C13type treatment with the 8 hpi group having undetectable levels of virus in most cases and less dramatic reductions observed in the 24 hpi group. Notably, one animal from the MP-12 treatment group and two from the placebo group succumbed prior to sacrifice on day 2, further demonstrating the severity of disease in animals that did not receive rMP12-C13type post-exposure vaccine treatment.

### Efficacy of Post-exposure Vaccination with rMP12-C13type in Hamsters Challenged i.n. with RVFV

**FIGURE 4:**
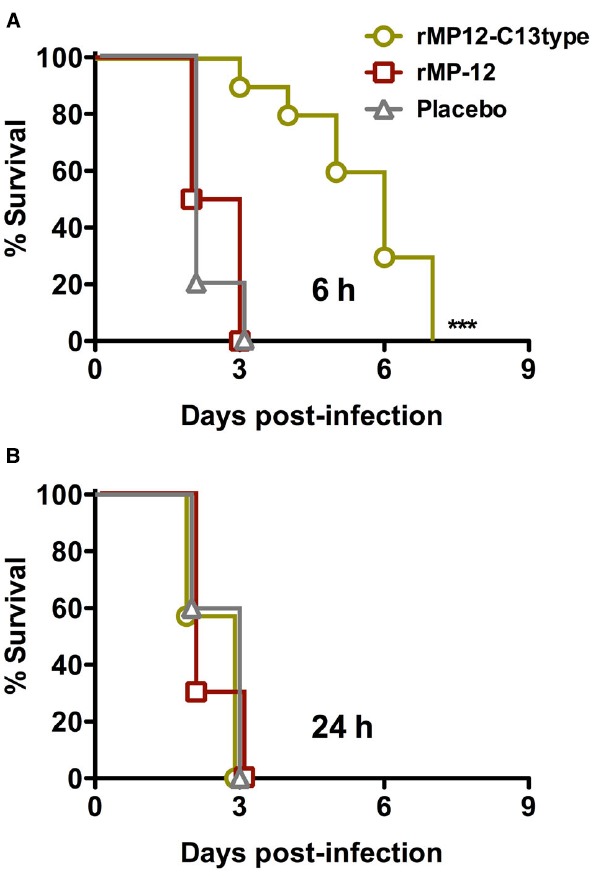
**Effect of post-exposure treatment with rMP12-C13type or rMP-12 vaccine strains on survival outcome in hamsters challenged i.n. with RVFV.** Animals in each group (*n* = 10) were treated s.c. with 1 × 10^5^ PFU of rMP12-C13type or MP-12 at (A) 6 h or (B) 24 h post-infection. ***P < 0.001 compared to animals treated with placebo.

Exposure to RVFV can also occur through the respiratory tract. Therefore, we also investigated the use of rMP12-C13type treatment in this capacity. In this study, a recombinant MP-12 virus (rMP-12) served as the comparison control for the rMP12-C13type vaccine, and along with the PBS placebo, all were administered by s.c. injection at 6 or 24 hpi with i.n. RVFV ZH501 to model respiratory tract exposure. As shown in Figure 4, only a weak protective effect based on extended survival time was observed in the i.n. challenge model in animals treated with rMP12-C13type within 6 hpi. There was no effect of rMP12-C13type treatment when administered 24 hpi (Figure 4). Considering the highly effective post-exposure vaccination observed in the s.c. challenge model, and the fact that rMP12-C13type treatment was more efficacious in the mouse i.n. infection model (Gowen et al., 2013), the results were unexpected.

### Measurement of Viral RNA Accumulation and IFN-β Induction Following Vaccination

**FIGURE 5:**
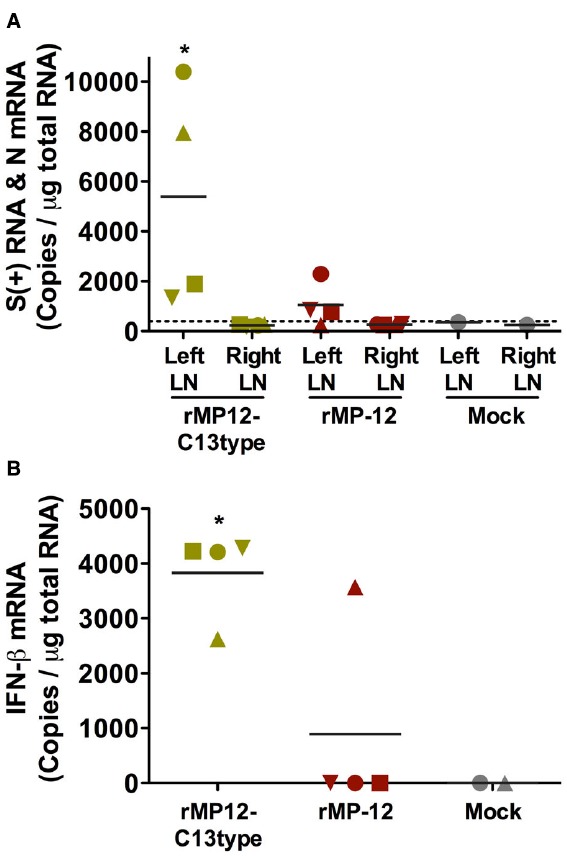
**Accumulation of RVFV S RNA and IFN-β mRNA in popliteal lymph nodes (LNs) of vaccinated hamsters.** Hamsters were vaccinated s.c. with 1 × 10^5^ PFU of rMP-12, rMP12-C13type or PBS (mock) in the left hind leg footpad. At 24 h post-vaccination, popliteal LNs were collected from the left and right hind legs. The RNA copies for (A) RVFV S RNA in the left and right popliteal LNs and for (B) IFN-β mRNA in the left popliteal LNs were measured by droplet digital PCR. Mean values are shown. Unique symbols represent the same animal across all parameters. The dashed line represents the limit of detection based on a mock-vaccinated control. *P < 0.05 compared to (A) the rMP12-C13type right LN and (B) rMP-12.

To gain insights into the mechanism by which the rMP12-C13type virus confers post-exposure protection, we measured RVFV S RNA and IFN-β mRNA at 24 h post-vaccination to understand the difference between rMP-12 and rMP12-C13type in terms of initial viral migration into draining LNs. Groups of hamsters (*n* = 4) were inoculated s.c. with 1 × 10^5^ PFU of rMP-12, rMP12-C13type or PBS (mock-inoculated controls) in the left hind footpad. At 24 h post-vaccination, popliteal LNs were collected from the left and right legs, and total RNA was extracted for copy number measurement of RVFV S RNA or IFN-β mRNA. The mean S RNA copies per microgram of total popliteal LN (left side) RNA from hamsters vaccinated with rMP-12 and rMP12-C13type were 1 × 10^3^ and 5.4 × 10^3^, respectively (Figure 5A). The respective contralateral popliteal LNs (right side) were also assayed as internal controls and found to have S RNA levels below the assay detection limits. Remarkably, only one of four hamsters from the rMP-12 group had detectable levels of IFN-β mRNA in the left popliteal LN, whereas all four from the rMP12-C13type group had >2.5 × 10^3^ copies per microgram of total RNA (Figure 5B). The mock-vaccinated hamster did not have detectable values for S RNA or IFN-β mRNA. Our results indicate that replication of the rMP12-C13type virus more consistently induces IFN-β gene expression compared to the rMP-12 virus at the draining LN.

## Discussion

Rodent models of severe viral diseases are important tools for the development of vaccines and therapeutics, as well as gaining insights into the mechanisms underlying disease pathogenesis. Mouse and hamster infection models are commonly used for proof-of-concept and early-stage preclinical drug and vaccine development (Smith et al., 2014). We previously reported on the efficacy of the rMP12-C13type vaccine virus when administered prophylactically after RVFV infection in mice (Gowen et al., 2013). Because the post-exposure therapeutic window was narrow (less than 1 h) and complete protection could not be achieved (only 30% survival) even when the rMP12-C13type vaccine was administered within 30 min of s.c. challenge with RVFV ZH501 (Gowen et al., 2013), we extended our investigation to include the hamster RVFV infection model. Unexpectedly, post-infection treatment with the same 1 × 10^5^ PFU dose of rMP12-C13type used in the mouse model studies conferred complete protection against uniformly lethal RVFV ZH501 challenge even when vaccination was delayed until 6 hpi. Remarkably, dosing with rMP12-C13type 12 hpi also provided a high level of protection (70% survival), and efficacy was still apparent when the vaccine was given 24 hpi.

In the mouse s.c. challenge study, the animals were inoculated with approximately 300 PFU of RVFV ZH501, with the placebo-treated mice having succumbed 4 ± 0.9 days post-infection (Gowen et al., 2013). In the present study, 30 PFU of virus was inoculated s.c. and the hamsters receiving placebo succumbed 2.4 ± 0.5 days following challenge. It is unclear why the rMP12-C13type vaccine was more effective against the more aggressive peracute disease caused by RVFV ZH501 infection in hamsters. Presumably there is greater and/or more rapid induction of type I IFN in vaccinated hamsters eliciting a more robust antiviral response. In mice, a direct comparison of induced type I IFN in serum and the presence of viral antigen at the draining LN were assessed following vaccination with rMP-12 and rMP12-C13type (Lihoradova et al., 2012). In addition to inducing greater levels of IFN-α, rMP12-C13type infection resulted in more efficient migration of dermal dendritic cells into popliteal LNs after s.c. footpad inoculation. In the present study, our results in hamsters showed the presence of rMP12-C13type viral RNA in draining popliteal LNs and more consistent induction of IFN-β mRNA compared to rMP-12. In the absence of NSs, rMP12-C13type virus replication probably triggers IFN-α/β through activation of RIG-I-like and other viral RNA-sensing cytosolic receptors that recognize viral RNA replication intermediates (Goubau et al., 2013). Our findings are consistent with the ineffectiveness of the MP-12 vaccine strain in the hamster model and suggest that the functional NSs protein produced by the MP-12 virus negatively affects the rapid induction of effective innate host antiviral defenses.

Another distinguishing feature observed with our evaluation of rMP12-C13type in the hamster model was that efficacy was greater in animals challenged with RVFV ZH501 by the s.c. route compared to i.n. route infection. In contrast, treatment of mice challenged i.n. with the ZH501 virus was substantially more effective than treatment of s.c.-infected animals (Gowen et al., 2013). These findings in mice, and our success with the initial post-exposure treatment studies resulting in complete protection of hamsters against s.c. inoculation of RVFV ZH501, heightened our expectations for post-exposure treatment of hamsters challenged by the i.n. route. In hindsight, the limited efficacy we observed when treating with rMP12-C13type 6 hpi suggests that a 30 min post-exposure treatment time was warranted and may have resulted in the 30% survival range observed in the mouse model (Gowen et al., 2013). One must also consider the fact that 1 × 10^5^ PFU of RVFV ZH501 was required to achieve lethality in hamsters by i.n. route infection. This higher challenge dose and direct access to the brain through the olfactory nerve may account for the reduced efficacy.

Collectively, our results shed light on the value of employing more than one species during initial efficacy studies and emphasizes the challenge of determining which model will be more predictive of outcome in humans or animals in the case of RVFV, which is also a significant livestock pathogen. The data presented also further supports the notion that despite the greater degree of attenuation with the rMP12-C13type vaccine virus, it retains its ability to replicate sufficiently to elicit protective adaptive immunity by a standard vaccination approach, with the added benefit of protecting against RVFV infection in a post-exposure setting through rapid induction of innate antiviral host defenses.

### Conflict of Interest Statement

The authors declare that the research was conducted in the absence of any commercial or financial relationships that could be construed as a potential conflict of interest.

## References

[B1] Bird B. H. Ksiazek T. G. Nichol S. T. Maclachlan N. J. (2009). Rift Valley fever virus. J. Am. Vet. Med. Assoc. 234, 883–893. 10.2460/javma.234.7.883 19335238

[B2] Borio L. Inglesby T. Peters C. J. Schmaljohn A. L. Hughes J. M. Jahrling P. B. (2002). Hemorrhagic fever viruses as biological weapons: medical and public health management. JAMA 287, 2391–2405. 10.1001/jama.287.18.2391 11988060

[B3] Caplen H. Peters C. J. Bishop D. H. (1985). Mutagen-directed attenuation of Rift Valley fever virus as a method for vaccine development. J. Gen. Virol. 66(Pt 10), 2271–2277. 10.1099/0022-1317-66-10-2271 4045430

[B4] Dauber B. Wolff T. (2009). Activation of the antiviral kinase PKR and viral countermeasures. Viruses 1, 523–544. 10.3390/v1030523 21994559 PMC3185532

[B5] Dungu B. Louw I. Lubisi A. Hunter P. Von Teichman B. F. Bouloy M. (2010). Evaluation of the efficacy and safety of the Rift Valley Fever Clone 13 vaccine in sheep. Vaccine 28, 4581–4587. 10.1016/j.vaccine.2010.04.085 20470792

[B6] Goubau D. Deddouche S. Reis e Sousa C. (2013). Cytosolic sensing of viruses. Immunity 38, 855–869. 10.1016/j.immuni.2013.05.007 23706667 PMC7111113

[B7] Gowen B. B. Bailey K. W. Scharton D. Vest Z. Westover J. B. Skirpstunas R. (2013). Post-exposure vaccination with MP-12 lacking NSs protects mice against lethal Rift Valley fever virus challenge. Antiviral Res. 98, 135–143. 10.1016/j.antiviral.2013.03.009 23523764 PMC3665270

[B8] Gowen B. B. Wong M. H. Jung K. H. Sanders A. B. Mendenhall M. Bailey K. W. (2007). In vitro and in vivo activities of T-705 against arenavirus and bunyavirus infections. Antimicrob. Agents Chemother. 51, 3168–3176. 10.1128/AAC.00356-07 17606691 PMC2043187

[B9] Habjan M. Pichlmair A. Elliott R. M. Overby A. K. Glatter T. Gstaiger M. (2009). NSs protein of Rift Valley fever virus induces the specific degradation of the double-stranded RNA-dependent protein kinase. J. Virol. 83, 4365–4375. 10.1128/JVI.02148-08 19211744 PMC2668506

[B10] Hunter P. Erasmus B. J. Vorster J. H. (2002). Teratogenicity of a mutagenised Rift Valley fever virus (MVP 12) in sheep. Onderstepoort J. Vet. Res. 69, 95–98. 12092782

[B11] Ikegami T. (2012). Molecular biology and genetic diversity of Rift Valley fever virus. Antiviral Res. 95, 293–310. 10.1016/j.antiviral.2012.06.001 22710362 PMC3586937

[B12] Ikegami T. Hill T. E. Smith J. K. Zhang L. Juelich T. L. Gong B. (2015). Rift Valley fever virus MP-12 vaccine is fully attenuated by a combination of partial attenuations in the S-, M- and L-segments. J. Virol. 89, 7262–7276. 10.1128/JVI.00135-15 25948740 PMC4473576

[B13] Ikegami T. Makino S. (2011). The pathogenesis of Rift Valley fever. Viruses 3, 493–519. 10.3390/v3050493 21666766 PMC3111045

[B14] Ikegami T. Narayanan K. Won S. Kamitani W. Peters C. J. Makino S. (2009). Rift Valley fever virus NSs protein promotes post-transcriptional downregulation of protein kinase PKR and inhibits eIF2alpha phosphorylation. PLoS Pathog. 5:e1000287. 10.1371/journal.ppat.1000287 19197350 PMC2629125

[B15] Ikegami T. Won S. Peters C. J. Makino S. (2006). Rescue of infectious Rift Valley fever virus entirely from cDNA, analysis of virus lacking the NSs gene, and expression of a foreign gene. J. Virol. 80, 2933–2940. 10.1128/JVI.80.6.2933-2940.2006 16501102 PMC1395455

[B16] Kainulainen M. Habjan M. Hubel P. Busch L. Lau S. Colinge J. (2014). Virulence factor NSs of Rift Valley fever virus recruits the F-box protein FBXO3 to degrade subunit p62 of general transcription factor TFIIH. J. Virol. 88, 3464–3473. 10.1128/JVI.02914-13 24403578 PMC3957945

[B17] Kalveram B. Lihoradova O. Ikegami T. (2011). NSs protein of Rift Valley fever virus promotes posttranslational downregulation of the TFIIH subunit p62. J. Virol. 85, 6234–6243. 10.1128/JVI.02255-10 21543505 PMC3126510

[B18] Kortekaas J. (2014). One Health approach to Rift Valley fever vaccine development. Antiviral Res. 106, 24–32. 10.1016/j.antiviral.2014.03.008 24681125

[B19] Laughlin L. W. Meegan J. M. Strausbaugh L. J. Morens D. M. Watten R. H. (1979). Epidemic Rift Valley fever in Egypt: observations of the spectrum of human illness. Trans. R. Soc. Trop. Med. Hyg. 73, 630–633. 10.1016/0035-9203(79)90006-3 575446

[B20] Le May N. Dubaele S. Proietti De Santis L. Billecocq A. Bouloy M. Egly J. M. (2004). TFIIH transcription factor, a target for the Rift Valley hemorrhagic fever virus. Cell 116, 541–550. 10.1016/S0092-8674(04)00132-1 14980221

[B21] Le May N. Mansuroglu Z. Leger P. Josse T. Blot G. Billecocq A. (2008). A SAP30 complex inhibits IFN-beta expression in Rift Valley fever virus infected cells. PLoS Pathog. 4:e13. 10.1371/journal.ppat.0040013 18225953 PMC2323286

[B22] Lihoradova O. Kalveram B. Indran S. V. Lokugamage N. Juelich T. L. Hill T. E. (2012). The dominant-negative inhibition of double-stranded RNA-dependent protein kinase PKR increases the efficacy of Rift Valley fever virus MP-12 vaccine. J. Virol. 86, 7650–7661. 10.1128/JVI.00778-12 22573861 PMC3416293

[B23] Madani T. A. Al-Mazrou Y. Y. Al-Jeffri M. H. Mishkhas A. A. Al-Rabeah A. M. Turkistani A. M. (2003). Rift Valley fever epidemic in Saudi Arabia: epidemiological, clinical, and laboratory characteristics. Clin. Infect. Dis. 37, 1084–1092. 10.1086/378747 14523773

[B24] McIntosh B. M. Russell D. Dos Santos I. Gear J. H. (1980). Rift Valley fever in humans in South Africa. S. Afr. Med. J. 58, 803–806. 7192434

[B25] Morrill J. C. Carpenter L. Taylor D. Ramsburg H. H. Quance J. Peters C. J. (1991). Further evaluation of a mutagen-attenuated Rift Valley fever vaccine in sheep. Vaccine 9, 35–41. 10.1016/0264-410X(91)90314-V 2008798

[B26] Morrill J. C. Jennings G. B. Caplen H. Turell M. J. Johnson A. J. Peters C. J. (1987). Pathogenicity and immunogenicity of a mutagen-attenuated Rift Valley fever virus immunogen in pregnant ewes. Am. J. Vet. Res. 48, 1042–1047. 3631685

[B27] Morrill J. C. Mebus C. A. Peters C. J. (1997a). Safety and efficacy of a mutagen-attenuated Rift Valley fever virus vaccine in cattle. Am. J. Vet. Res. 58, 1104–1109. 9328662

[B28] Morrill J. C. Mebus C. A. Peters C. J. (1997b). Safety of a mutagen-attenuated Rift Valley fever virus vaccine in fetal and neonatal bovids. Am. J. Vet. Res. 58, 1110–1114. 9328663

[B29] Morrill J. C. Peters C. J. (2003). Pathogenicity and neurovirulence of a mutagen-attenuated Rift Valley fever vaccine in rhesus monkeys. Vaccine 21, 2994–3002. 10.1016/S0264-410X(03)00131-2 12798643

[B30] Muller R. Saluzzo J. F. Lopez N. Dreier T. Turell M. Smith J. (1995). Characterization of clone 13, a naturally attenuated avirulent isolate of Rift Valley fever virus, which is altered in the small segment. Am. J. Trop. Med. Hyg. 53, 405–411. 7485695 10.4269/ajtmh.1995.53.405

[B31] Reed L. J. Muench H. (1938). A simple method of estimating fifty percent endpoints. Am. J. Hyg. 27, 493–497.

[B32] Scharton D. Bailey K. W. Vest Z. Westover J. B. Kumaki Y. Van Wettere A. (2014). Favipiravir (T-705) protects against peracute Rift Valley fever virus infection and reduces delayed-onset neurologic disease observed with ribavirin treatment. Antiviral Res. 104, 84–92. 10.1016/j.antiviral.2014.01.016 24486952 PMC3975078

[B33] Smith D. R. Holbrook M. R. Gowen B. B. (2014). Animal models of viral hemorrhagic fever. Antiviral Res. 112C, 59–79. 10.1016/j.antiviral.2014.10.001 25448088

[B34] von Teichman B. Engelbrecht A. Zulu G. Dungu B. Pardini A. Bouloy M. (2011). Safety and efficacy of Rift Valley fever Smithburn and Clone 13 vaccines in calves. Vaccine 29, 5771–5777. 10.1016/j.vaccine.2011.05.055 21664400

[B35] Wilson W. C. Bawa B. Drolet B. S. Lehiy C. Faburay B. Jasperson D. C. (2014). Evaluation of lamb and calf responses to Rift Valley fever MP-12 vaccination. Vet. Microbiol. 172, 44–50. 10.1016/j.vetmic.2014.04.007 24856133

